# Assessment of Antidiabetic Activity of the Shikonin by Allosteric Inhibition of Protein-Tyrosine Phosphatase 1B (PTP1B) Using State of Art: An In Silico and In Vitro Tactics

**DOI:** 10.3390/molecules26133996

**Published:** 2021-06-30

**Authors:** Mohd Saeed, Ambreen Shoaib, Munazzah Tasleem, Nadiyah M. Alabdallah, Md Jahoor Alam, Zeina El Asmar, Qazi Mohammad Sajid Jamal, Fevzi Bardakci, Saad S. Alqahtani, Irfan Ahmad Ansari, Riadh Badraoui

**Affiliations:** 1 Department of Biology, College of Sciences, University of Hail, Hail 81451, Saudi Arabia; j.alam@uoh.edu.sa (M.J.A.); z.elasmar@uoh.edu.sa (Z.E.A.); fbardakci@adu.edu.tr (F.B.); badraouir@yahoo.fr (R.B.); 2 Department of Clinical Pharmacy, College of Pharmacy, Jazan University, P.O. Box No. 114, Jazan 45142, Saudi Arabia; ssalqahtani@jazanu.edu.sa; 3 School of Electronic Science and Engineering, University of Electronic Science and Technology of China, Chengdu 610054, China; munazzah.t@gmail.com; 4 Department of Biology, College of Science, Imam Abdulrahman Bin Faisal University, 383, Dammam 31113, Saudi Arabia; nmalabdallah@iau.edu.sa; 5 Department of Health Informatics, College of Public Health and Health Informatics, Qassim University, Al Bukayriyah 52571, Saudi Arabia; m.quazi@qu.edu.sa; 6 Department of Biosciences, Integral University, Lucknow 226026, India; ahmadirfan.amu@gmail.com; 7 Section of Histology-Cytology, Medicine Faculty of Tunis, University of Tunis El Manar, La Rabta-Tunis 1007, Tunisia

**Keywords:** diabetes mellitus, protein-tyrosine phosphatase, shikonin, hypoglycemic, molecular docking

## Abstract

*Diabetes mellitus* is a multifactorial disease that affects both developing and developed countries and is a major public health concern. Many synthetic drugs are available in the market, which counteracts the associated pathologies. However, due to the propensity of side effects, there is an unmet need for the investigation of safe and effective drugs. This research aims to find a novel phytoconstituent having diminished action on blood glucose levels with the least side effects. Shikonin is a naturally occurring naphthoquinone dying pigment obtained by the roots of the *Boraginaceae* family. Besides its use as pigments, it can be used as an antimicrobial, anti-inflammatory, and anti-tumor agent. This research aimed to hypothesize the physicochemical and phytochemical properties of Shikonin’s in silico interaction with protein tyrosine phosphate 1B, as well as it’s in vitro studies, in order to determine its potential anti-diabetic impact. To do so, molecular docking experiments with target proteins were conducted to assess their anti-diabetic ability. Analyzing associations with corresponding amino acids revealed the significant molecular interactions between Shikonin and diabetes-related target proteins. In silico pharmacokinetics and toxicity profile of Shikonin using ADMET Descriptor, Toxicity Prediction, and Calculate Molecular Properties tools from Biovia Discovery Studio v4.5. Filter by Lipinski and Veber Rule’s module from Biovia Discovery Studio v4.5 was applied to assess the drug-likeness of Shikonin. The in vitro studies exposed that Shikonin shows an inhibitory potential against the PTP1B with an IC50 value of 15.51 µM. The kinetics studies revealed that it has a competitive inhibitory effect (Ki = 7.5 M) on the enzyme system, which could be useful in the production of preventive and therapeutic agents. The findings of this research suggested that the Shikonin could be used as an anti-diabetic agent and can be used as a novel source for drug delivery.

## 1. Introduction

*Diabetes mellitus* (DM) can be described as a multi metabolic dysfunction ascribable to either absolute or relative insulin deficiency leading to hyperglycemia [1]. Specialists have prognosticated that incidence may accumulate to approximately 64% by 2025 and may target 53.1 million people [2]. In 2010, the estimated global prevalence of diabetes among adults was 285 million (6.4%), with the number projected to rise to about 439 million by 2030 (7.7%) [3]. The ratio of people with type 2 diabetes is increasing at a higher pace globally. The majority of such can be found in underdeveloped and developing countries. Perhaps around 463 million of the adult population is suffering from diabetes, and it may reach about 700 million by 2045. Saudi Arabia is the most extensive kingdom in the Middle East, occupying approximately four-fifths of the Arabian Peninsula, with a population of around 33.3 million [4]. The pace of DM in the adult population in Saudi Arabia is increasing ubiquitously. Around one-fourth of the adult human population in the country is suffering from DM, and it is expected to increase manifold by 2030 [5].

The enzymes that play an essential role in type 2 DM are PTP-1B, glucokinase, dipeptidyl peptidase-IV, peroxisome proliferator-activated receptor, aldose reductase, insulin receptor, etc. [6]. PTP-1B is a distinctive enzyme that plays a substantial part in insulin signaling pathways that are implicated in type 2 diabetes and associated metabolic disorders as a negative regulator [7]. This functions by showing improvement in insulin resistance and normalizing insulin and plasma glucose levels without triggering hypoglycemia. PTPs are characterized by a preserved active site succession, in which the cysteine residue plays a role as a nucleophile, which is fundamental for catalysis reactions. There are 107 PTP genes and among all, PTP1B has the same homogeneity from human placental tissue [8].

PTPs are optimistic molecular markers for diabetes care. However, no PTP inhibitor that is appropriate for clinical use has yet to be discovered [9]. PTP1B has received the most attention in research relating to the production of novel anti-diabetic drugs, even though many PTPs have been implicated in diabetes [10,11]. Thus, the present research work was designed to evaluate the antidiabetic activity of a naturally occurring pigment Shikonin. The present study worked on docking, virtual screening, molecular dynamics, in vitro inhibitory, and kinetics assay on PTP1B.

## 2. Materials and Methods

### 2.1. Chemicals, Reagents, and Cell Culture

Sigma-Aldrich Co. supported Shikonin and p-nitrophenyl phosphate (pNPP) (St. Louis, MO, USA). Biomol International LP provided the Protein Tyrosine Phosphate 1B (PTP1B; human recombinant) (Plymouth Meeting, PA, USA). All other chemicals and solvents were purchased from industrial sources and were of reagent grade.

### 2.2. In Silico Docking Studies of Shikonin

The ligand Shikonin (5,8-dihydroxy-2-[(1R)-1-hydroxy-4-methylpent-3-enyl] naphthalene-1,4-dione) is obtained from PubChem [12]. The X-ray crystallographic structure of the target protein, protein-tyrosine phosphatase 1B (PTP1B) (PDB ID: 1AAX), refined at a1.9-Å resolution, was repossessed from RCSB Protein Data Bank. 1AAX is bound to bis-(para-phosphophenyl) methane (BPPM) in its binding pocket [13]. The presence of domains in the receptor was identified using SMART [14] and ScanProsite [15] online tools. Docking studies using the GOLD suite [16] started with protein preparation by adding hydrogen atoms and removing water molecules. All ligands were removed and a cavity around the binding site was generated by adjusting a radius of 9Å in X, Y, and Z directions in place of BPPM. A Gold Fitness Score was selected to evaluate and rank the predicted ligand conformations for optimal prediction of the best pose after accurate binding conformation. The ligand pose with the highest Gold Fitness Score was selected for intra-molecular interaction studies.

### 2.3. Identification of Compounds Similar to Shikonin 

To identify compounds with similar features as that of Shikonin to inhibit PTP1B, pharmacophore modeling, virtual screening, molecular docking, and ADMET approaches were carried out.

#### 2.3.1. Pharmacophore Modeling and Virtual Screening

A pharmacophore defines a molecule’s spatial arrangement and essential features with the same biological interactions, such as hydrogen bond, hydrophobic, charged, or aromatic features with a specific biological target to initiate a biological response. Computer-aided drug design techniques such as pharmacophore modeling, virtual screening, molecular docking, and molecular dynamic simulation are important techniques for discovering novel hits/leads against any biologically active macromolecule [17,18,19]. Ligand-based pharmacophore modeling was applied to identify Shikonin-derived compounds with similar features against the PTP1B structure (PDB ID: 1AAX). Shikonin-derived compounds were obtained based on the formed intra-molecular interactions between PTP1B and Shikonin. Based on the Shikonin-1AAX docked complex, pharmacophore features were generated with the 2-hydrogen bond donor, 1-hydrogen bond acceptor, 1-aromatic, and 1-hydrophobic feature to screen more diverse small molecules from the Zinc Natural Products database using ZINCPharmer [20].

To identify potential small molecules from the large database of molecules that bind to the therapeutic target, virtual screening techniques are used for drug discovery to deliver hits in a time and cost-effective way [21]. Furthermore, to carry out virtual screening, pharmacokinetic and pharmacodynamic analysis, the screening result of small molecules from ZINCPharmer was retrieved in anSDF file format.

The structural similarity of known and unknown compounds with known active small molecules is evaluated using a ligand-based screening method that includes pharmacophore modeling to find the best ligands with the best activity against the therapeutic target [22]. However, to envisage the ligand’s optimum binding conformation and attraction with the target protein, structure-based virtual screening such as molecular docking is applied. Identified compounds through ZINCPharmer were subjected to virtual screening using the GOLD suite to evaluate the binding affinity with PTP1B. The top 100 compounds with the highest Gold Fitness Score were selected for drug-likeness and ADMET analysis.

#### 2.3.2. Drug Likeness and In-Silico ADMET Prediction

In silico technology facilitates experimental drug trials by the identification of lead molecules to improve the success rate. For example, the pharmacokinetic properties, ADMET, are used to predict oral bioavailability, cell-permeation, metabolism, and removal of small molecules, thus assisting in understanding the activity of asmall molecule within the human body. ADME-Toxicity of Shikonin was measured using the ‘ADMET Descriptor’ module of Biovia Discovery Studio v4.5 software.Oral bioavailability, blood-brain barrier intrusion (BBB), cytochrome P450 2D6 (CYP2D6) induction, nephrotoxicity, host gut absorption, and serum protein binding were all predicted by theoretical equations [23].The top 100 molecules with the highest Gold Fitness Scorewere also screened for pharmacokinetics and pharmacological properties using the ADMET Descriptor, Toxicity Prediction, and Calculate Molecular Properties tools from Biovia Discovery Studio v4.5. The ‘Filter by Lipinski and Veber Rule’ module from Biovia Discovery Studio v4.5 was applied to assess the drug-likeness of Shikonin and top 100 molecules to determine the level of drug safety and efficacy for administration purposes. The top filtered molecules were docked with 1AAX in its binding pocket by replacing the co-crystal ligand-BPPM using the GOLD Suite to explore intra-molecular interaction using the ‘View Interactions’ tool from Biovia Discovery Studio v4.5.

#### 2.3.3. Molecular-Dynamics Simulation Studies

Molecular Dynamics (MD) simulations of the protein-ligand complex were performed using the Desmond application available with Schrodinger maestro to study the dynamic activity of PTP1B in simulated physiological conditions. The protein-ligand complex (33,409 atoms) was solvated in a TIP3P water-filled 10 10 10 orthorhombic periodic boxes [24]. By adding a sufficient number of 5 Na counterions, the entire mechanism was neutralized. Using OPLS 2005 as a force-field, this solvated device was energy minimized and location restrained [24]. Furthermore, a 100-ns MD run at 1 atm pressure and 300 K temperature was performed using the NPT ensemble with a recording interval of 100 ps, yielding 1000 reading frames. To check the stability, compactness, structural fluctuations, and protein-ligand interactions in a solvated system, various parameters of the MD simulation research, such as ligand binding site analysis, root mean square deviation (RMSD), root mean square fluctuation (RMSF), protein-ligand (PL) contacts, secondary structure element (SSE) analysis, and so on were analyzed.

### 2.4. In Vitro Anti-Diabetic Activity

#### 2.4.1. Protein Tyrosine Phosphate 1B (PTP1B) Inhibitory Assay

pNPP was used to test the inhibitory function of PTP1B. In a plate with or without a sample, a recombinant PTP1B enzyme (0.5 units diluted in PTP1B reaction buffer) was added. The plate was pre-incubated for 10 minutes at 37 °C before adding the substrate (2 mMpNPP). The enzymatic reaction was stopped by adding 10 M NaOH after 15 minutes of incubation at 37 °C. A microplate spectrophotometer was used to calculate the absorbance at 405 nm (Molecular Devices, Sunnyvale, CA, USA). The reference compound was ursolic acid [25]. The results were expressed as mean ± S.E.M. of three independent experiments performed in duplicate.

#### 2.4.2. Kinetic Study Against PTP1B

Two kinetic techniques, Lineweaver–Burk and Dixon plots, were used in tandem to figure out the kinetic process [25]. The enzymatic inhibition by the Shikonin was evaluated by monitoring the effects of different substrate concentrations (5, 10, 15, 20, and 25 mMpNPP for PTP1B in the Dixon plot) to determine the modes of enzyme inhibition (single reciprocal plot). In the presence of different concentrations of Shikonin (0, 2, 4, 8, and 16 M), a Lineweaver–Burk plot (double reciprocal plot) for the inhibition of PTP1B was obtained. The inhibition constant (Ki) was calculated by interpreting the Dixon plot, in which the x-axis value indicates −Ki.

### 2.5. Statistical Analysis

The statistical significance was determined using aone-way analysis of variance (ANOVA) and the student’s *t*-test (Systat Inc., Evanston, IL, USA). A *p*-value of less than 0.01 was deemed meaningful. The mean and standard error of the mean is used to display all of the data (SEM).

## 3. Results and Discussion

### 3.1. In Silico Docking Studies of Shikonin

**Figure 1:**
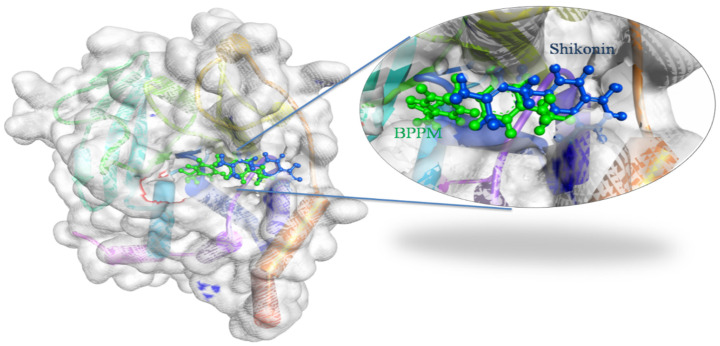
Docking view of Shikonin in the binding sites of PTP1B (PDB ID: 1AAX). On the left side is the surface view of the docked complex-Shikonin and 1AAX, on the right side, a binding pocket is magnified to present Shikonin (shown in the blue-colored ball and stick view) superimposed on the co-crystal ligand-BPPM (shown in the green-colored ball and stick view). Diagrams are prepared in the Biovia Discovery Studio.

The docking results of Shikonin and 1AAX provided optimum information for the binding orientation and ligand-receptor interactions. Shikonin was observed to dock in the same binding pocket of 1AAX as reported in the RCSB Protein Data Bank. A total of 10 conformations of Shikonin were obtained, and the best conformation with the highest GOLD Fitness Score of 46.31 was selected for further studies. The best conformation of Shikonin is superimposed on the co-crystal ligand BPPM in the PTP1B structure, Figure 1.

**Figure 2:**
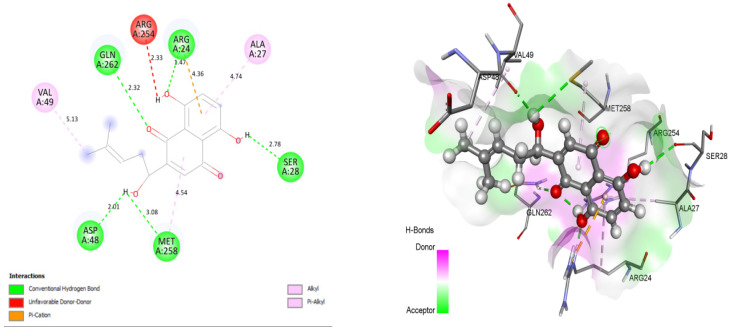
Docking view of Shikonin in the binding sites of PTP1B (PDB ID: 1AAX). On the left side is the stereo view of the docked complex-Shikonin and 1AAX, and on the right side are Shikonin-1AAX interactions presented in 2D. Diagrams are drawn in Discovery Studio, with hydrogenbonds shown as agreen dashed line. Protein structure residues are annotated with a 3-letter amino acid code, while Shikonin is presented in a ball-and-stick style.

Close intra-molecular interactions formed by Shikonin within the binding pocket of 1AAX include the hydrogen bond formation with ARG24, GLN262, ASP48, MET258, and SER28. Hydrophobic interactions including alkyl and pi-alkyl interaction were formed with residues VAL49, MET258, ARG24, and ALA27, as shown in Figure 2. Common intra-molecular interactions formed by BPPM and Shikonin in the binding pocket of 1AAX revealed the presence of H-bonds with active site residues ARG24, and GLN262, indicating their vital role in forming stable complexes.

### 3.2. Pharmacophore Modeling and Virtual Screening

**Figure 3:**
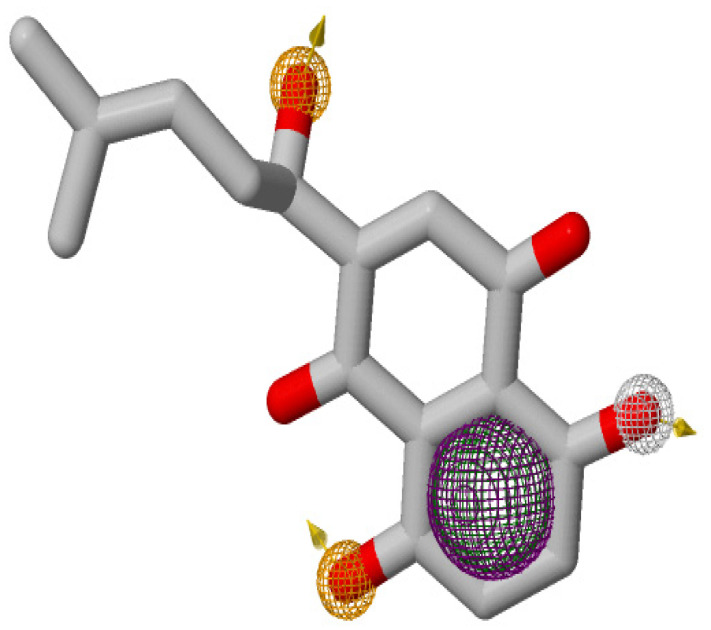
Pharmacophore features generated for Shikonin on ZINCPharmer. The hydrophobic, hydrogen bond donor, and hydrogen bond acceptors, and aromatic features are displayed in mesh spheres of green, white, orange, and purple, respectively. The orange arrows indicate the constraint direction.

Ligand-based virtual screening performed with selected pharmacophore features of Shikonin yielded 1860 compounds from the ZINC Natural Product database. Selected pharmacophore features of Shikonin for ligand-based virtual screening using ZINCPharmerare shown in Figure 3. Virtual screening was performed by the GOLD suite on all of the 1860 compounds within the binding pocket of the PTP1B structure, which resulted in various confirmations. The Gold Fitness Score for the best conformation of each ligand ranges from 145.42 to 33.23.

### 3.3. Drug Likeness and In Silico ADMET Prediction

**Figure 4:**
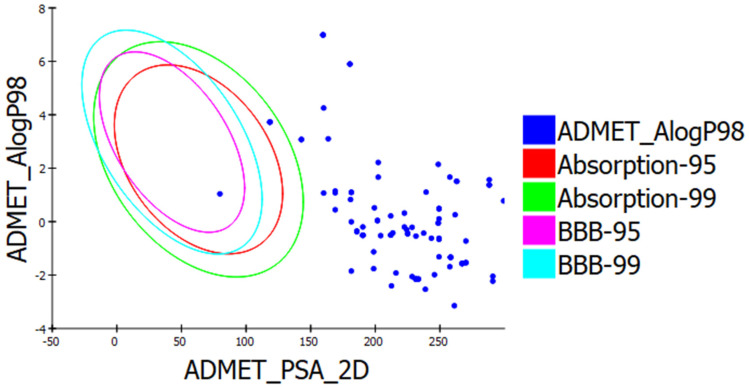
ALogP versus polar surface area (PSA) plot for Shikonin pharmacophores showing the 95% and 99% confidence limit ellipse corresponding to the blood-brain barrier (BBB) and intestinal absorption.

A total of 100 compounds with the best Gold Fitness Score were selected for pharmacokinetic, pharmacodynamic, and drug-likeness analysis. [26]. The ADME descriptors of Shikonin and the top 100 docked compounds were calculated for drug-likeness studies. Pharmacokinetic screening of the top 100 compounds revealed that ZINC31168554 passed Lipinski’s rule of five for oral bioavailability. TheADME model using descriptors 2D PSA and AlogP98 was developed to compute intestinal absorption and blood-brain barrier penetration of the compounds based on 95% and 99% confidence ellipses. The ADME Descriptor screening revealed that ZINC31168554 has 99% confidence levels for human gut and BBB penetration, as shown in Table S1 [26]. Figure 4 shows the plot of the ALogP and polar surface area for the top 100 compounds.

The United States Food and Drug Administration (USFDA,) TOPKAT software from Biovia Discovery Studio was utilized for predicting compounds with toxicity risk. The top 100 compounds were assessed for toxicity and found to have no chance of carcinogenic effects, teratogenicity, certain compounds were found to possess no ocular sensitivity, or skin rashes, however, compounds were found to possess developmental toxicity and mild to severe skin and ocular irritancy, as shown in Table S2. The top 100 compounds were filtered by the Lipinski and Veber’s Rules using Biovia Discovery Studio. The compound- ZINC31168041, ZINC31168045, ZINC31168041, and ZINC31168554 were passed through the Lipinski and Veberrules filter. Therefore, these compounds were further studied for docking and intra-molecular interaction studies. The ADME screening results and toxicity prediction values of these molecules are summarized in Table 1, Table 2 and Table 3. All ADMET values obtained for Shikonin were found within an acceptable range. The intestinal absorption level for Shikonin was found to be 0, indicative of good absorption. Good aqueous solubility of –2.712 at level 3 was predicted using linear regression in water at 25 °C along with good human intestinal absorption. Calculated values of blood-brain penetration were found to be –0.93, indicative of low penetration of Shikonin after oral administration. Cytochrome P450 2D6 (CYP2D6) enzyme inhibition of Shikonin was observed to be false. However, hepatotoxicity of Shikonin was observed to be true. Other filtered compounds show good aqueous solubility and no hepatotoxicity except for ZINC31168554 however, they poorly bind to plasma protein. The filtered compounds and Shikonin violets only possess higher TPSA values, indicating that these compounds have low oral bioavailability.

### 3.4. Docking and Intra-Molecular Interaction Analysis of Top 4 Compounds

The top 4 filtered compounds were docked in the binding pocket of PTP1B using GOLD Suite. Among all the compounds, ZINC31168045 was found to possess the highest GoldFitness Score of 118.114. Intra-molecular interaction studies of these compounds described the role of vital active site residues involved in forming hydrogen bonds. ARG24 forms hydrogen bonds with Shikonin, ZINC31168041, ZINC31168045, and ZINC31168048. SER216 forms hydrogen bonds with ZINC31168041, ZINC31168045, and ZINC31168554. GLN266 forms hydrogen bonds with ZINC31168554, ZINC31168045, and ZINC31168048. ARG254 forms hydrogen bonds with ZINC31168041, ZINC31168045, and ZINC31168048. SER215 and ARG221 form hydrogen bonds with all the top 4 filtered compounds as shown in Table 4.

**Figure 5:**
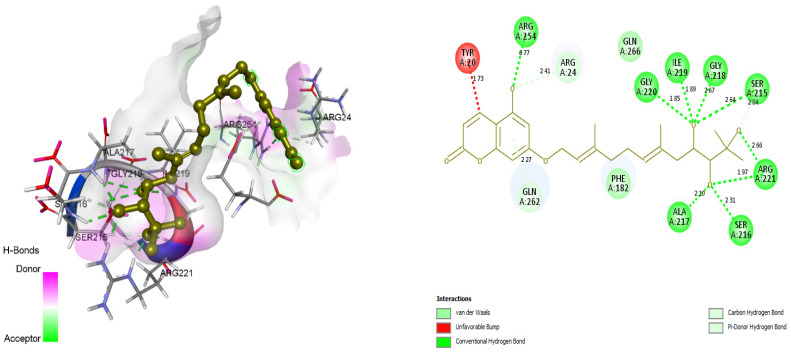
Docking view of ZINC31168041in the binding sites of PTP1B (PDB ID: 1AAX). On the left side is the stereo view of the docked complex-ZINC31168041 and 1AAX, on the right side, ZINC31168041-1AAX interactions are presented in 2D view. Diagrams are drawn in Discovery Studio, hydrogen-bonds are shown as agreen-dashed line along with the distance in Å. Protein structure residues are annotated with a 3-letter amino acid code, while ZINC31168041 is presented in agreen-colored ball and stick style.

**Figure 6:**
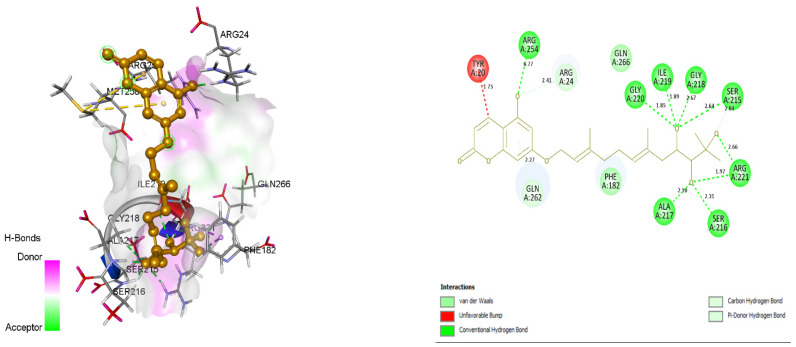
Docking view of ZINC31168045 in the binding sites of PTP1B (PDB ID: 1AAX). On the left side is the stereo view of the docked complex-ZINC31168045 and 1AAX, on the right side ZINC31168045 -1AAX interactions are presented in 2D. Diagrams are drawn in Discovery Studio, hydrogen-bonds are shown in a green-dashed line along with the distance in Å. Protein structure residues are annotated with a 3-letter amino acid code, while ZINC31168045 is presented in anorange-colored ball and stick style.

**Figure 7:**
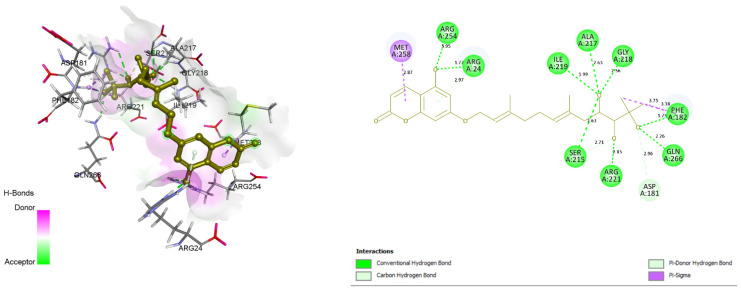
Docking view of ZINC31168048 in the binding sites of PTP1B (PDB ID: 1AAX). On the left side is the stereo view of the docked complex-ZINC31168048 and 1AAX, and on the right side ZINC31168048 -1AAX interactions are presented in 2D. Diagrams are drawn in Discovery Studio, hydrogen-bonds are shown as a green-dashed line along with the distance in Å. Protein structure residues are annotated with a 3-letter amino acid code, while ZINC31168048 is presented in agreen-colored ball and stick style.

**Figure 8:**
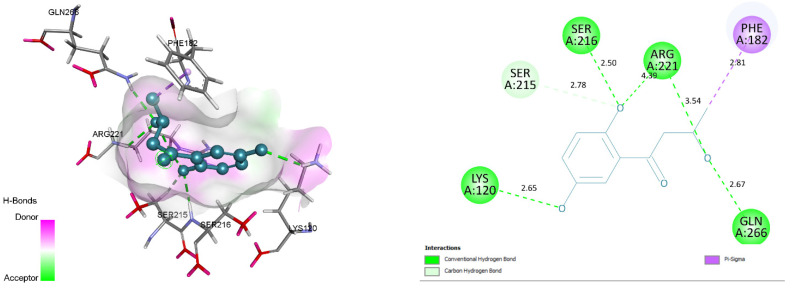
Docking view of ZINC31168554 in the binding sites of PTP1B (PDB ID: 1AAX). On theleft side is the stereo view of the docked complex- ZINC31168554 and 1AAX, on the right side ZINC31168554 -1AAX interactions are presented in 2D. Diagrams are drawn in Discovery Studio, hydrogen-bonds are shown as a green-dashed line along with the distance in Å. Protein structure residues are annotated with a 3-letter amino acid code, while ZINC31168554 is presented in ablue-colored ball and stick style.

Conserved active site residues forming H-bonds with filtered compounds and the co-crystal ligand BPPM are ARG24, SER215, SER216, ARG221, and ARG254. 3D and 2D docking pose of the filtered compounds on the PTP1B receptor are shown in Figure 5, Figure 6, Figure 7 and Figure 8. The receptor protein (PDB ID: 1AAX) was further investigated for the presence of domain and motifs using ScanProsite and SMART. Two highly conserved domains, ‘TYR_PHOSPHATASE_PTP’ with a score of 52.411 that lies between 3 and 277 residues, and ‘TYR_PHOSPHATASE_2’ domain with a score of 16.141 that lies within 189 to 268 residues are observed in 1AAX. Tyrosine-specific protein phosphatases play an essential role in removing a phosphate group attached to a tyrosine residue to control cell growth, proliferation, differentiation, and transformation. Furthermore, protein tyrosine phosphatases are characterized by the PTP signature motif (I/V)HCXAGXGR(S/T), including two essential residues CYS and ARG, for catalysis [27]. Therefore, all the identified residues forming H-bonds and hydrophobic interactions lie within the conserved domains. In addition, ARG 221 is an essential residue of the PTP signature motif.

### 3.5. Molecular Dynamic Simulation Studies

Desmond 6.1 was used to run molecular dynamics simulations of the protein-ligand complex (Maestro v12.3). Since the MD simulation offers information about the receptor-ligand complex over time, we ran it on the complex for 100 ns after docking. We evaluated the trajectory files for root mean square deviation (RMSD), root mean square fluctuation (RMSF), protein-ligand interactions, and other factors after the simulation.

**Figure 9:**
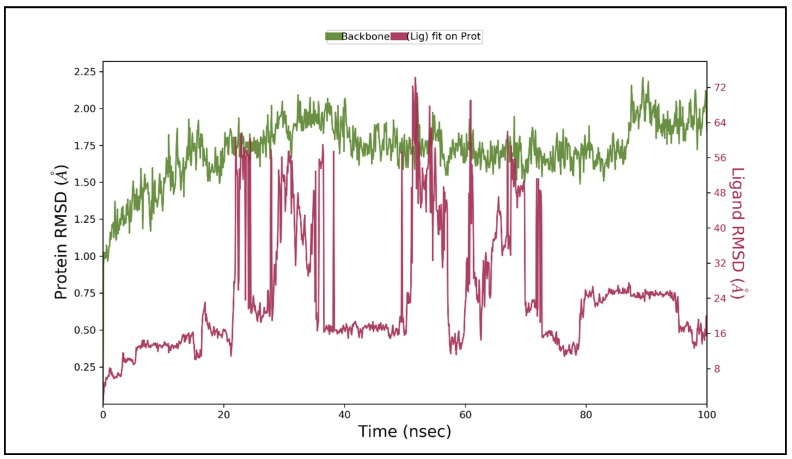
Root means square deviation (RMSD) of protein and ligand after the initial RMSD values were stabilized. This plot shows RMSD values for protein on the left Y-axis, whereas for ligand, these values are indicated on the right Y-axis. The RMSD graph for the backbone is shown in green, and for theligand fit on protein in red.

The mean variation in the displacement of an atom in a particular molecular conformation compared to a reference conformation is defined by the root mean square deviation (RMSD). The RMSD of each frame conformation residue relative to the average conformation is used to calculate the versatility of a protein region. In trajectory analysis, the complex RMSD found within the range of 2.02 Å while stabilizing the structure for 100 ns of simulation. The initial RMSD value of the complex was 0.95 Å. RMSD values fluctuated but started stabilizing after 30 ns, which then increased the maximum at 2.02 Å. The RMSD of ligand fit on the protein was almost constant at 1.99 Å from 30ns onwards, implying a stable structure. The cohesion, uniformity, structural variability, and protein-ligand interactions in a solvated system were studied as the backbone atoms at 1.99 Å. The backbone RMSD of 1.99 Å is shown in Figure 9.

**Figure 10:**
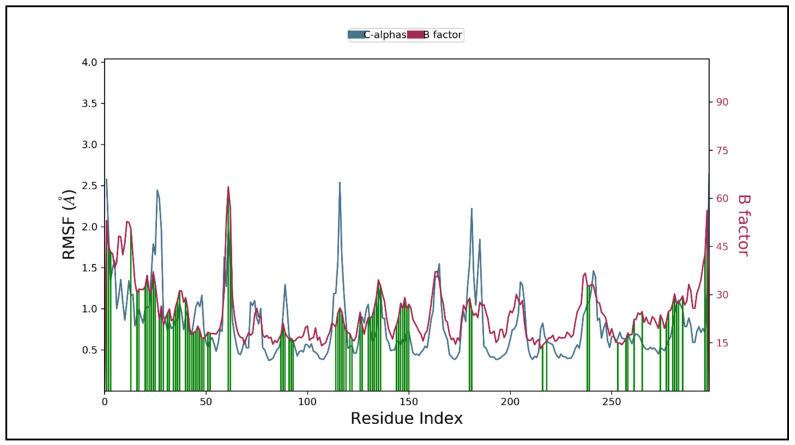
The RMSF protein backbone and ligand complex, the red color shows the B factor, which means the PDB and green shows the interaction of the ligand to the protein.

To illustrate the general changes along the protein chain, RMSF was computed throughout the simulation.The peaks in the RMSF plot indicate regions of fluctuation during the simulation, as shown in Figure 10. 

**Figure 11:**
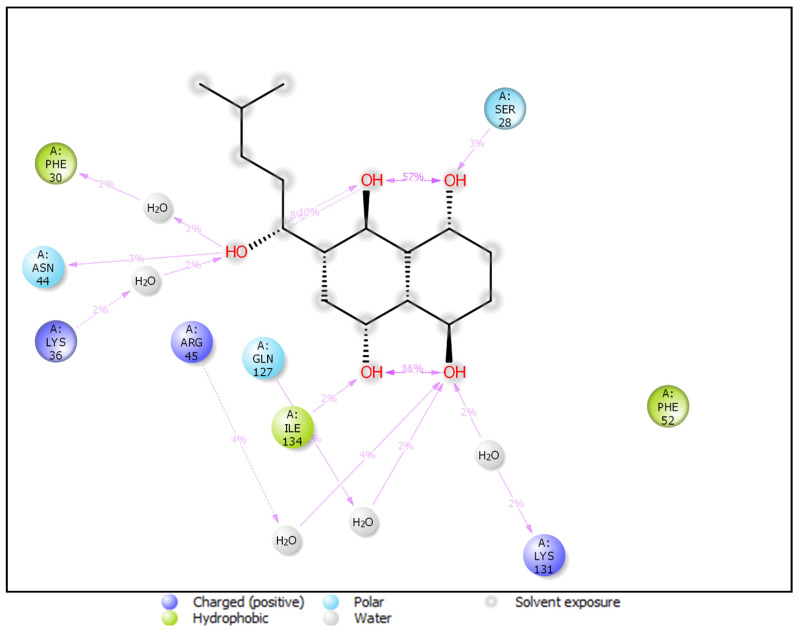
Protein-ligand complex interaction during the molecular docking simulation.

The protein-ligand complex was found less flexible however, the RMSF plot indicates variations in certain regions. During the simulation, ASN44, ILE134, and SER28 form hydrogen bond backbone interactions, while PHE30, LYS36, ARG45, GLN127, and LYS131 form hydrogen bond sidechain interactions. There are five water molecules directly involved in the hydrogen bond to the backbone of the protein-ligand complex. PHE30 and ILE134 form hydrophobic interactions while the backbone interactions LYS131, LYS36, and ARG45, participate in positively charged atom interactions. ASN44, GLN127, and SER28 are involved in forming polar molecular interactions (Figure 11).

A 100 ns MD simulation was performed to investigate protein-ligand stability, robustness, heterogeneity, and residue interactions. Our observations revealed an increase in the stability in the presence of the ligand. After 30 s, the RMSD of lig fit on protein begins to stabilize, and after 100 s, the observed RMSD was 1.99. During the simulation time, a number of hydrogen bonds, hydrophobic interactions, and water bridges were formed. Our findings strongly suggest that the detected molecule may be a possible lead molecule for inhibiting the actions of the proteins by interacting at the binding pocket.

### 3.6. Inhibitory Activity of Shikonin against PTP1B

**Figure 12:**
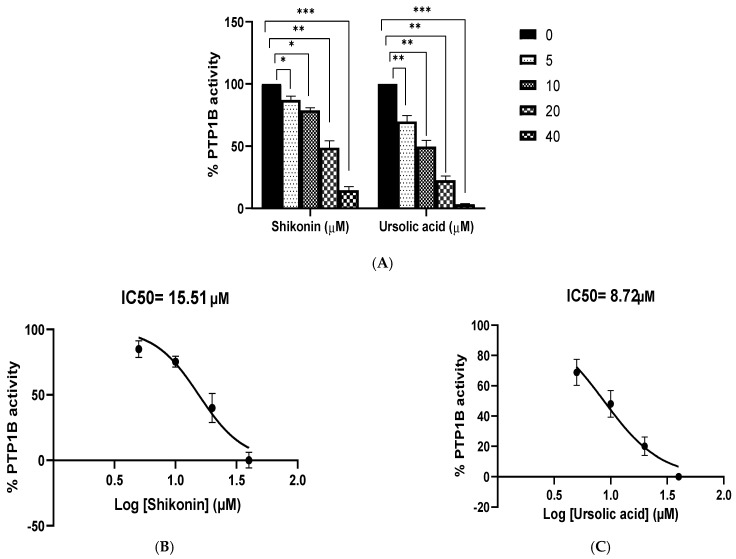
(**A**) Dose-dependent protein tyrosine phosphatases 1B inhibitory activity of Shikonin. Ursolic acid, a strong inhibitor of PTP1B, was used as a positive control. The data shown are mean ± S.E.M. of three independent experiments performed in duplicate (* *p* < 0.01, ** *p* < 0.001, and *** *p* < 0.0001 represent a significant difference compared with the control). (**B**) IC50 graph of Shikonin against PTP1B. (**C**) IC50 graph of ursolic acid against PTP1B.

Shikonin’s inhibitory properties against PTP1B were tested using p-nitrophenyl phosphate (pNPP) as a substrate. Figure 12A–C shows that the Shikonin had a powerful inhibitory effect on PTP1B, with an IC50 of 15.51 M. Ursolic acid, which served as a positive control, inhibited PTP1B with an IC50 of 8.72 M.

### 3.7. Enzyme Kinetics of PTP1B Inhibition by Shikonin

**Figure 13:**
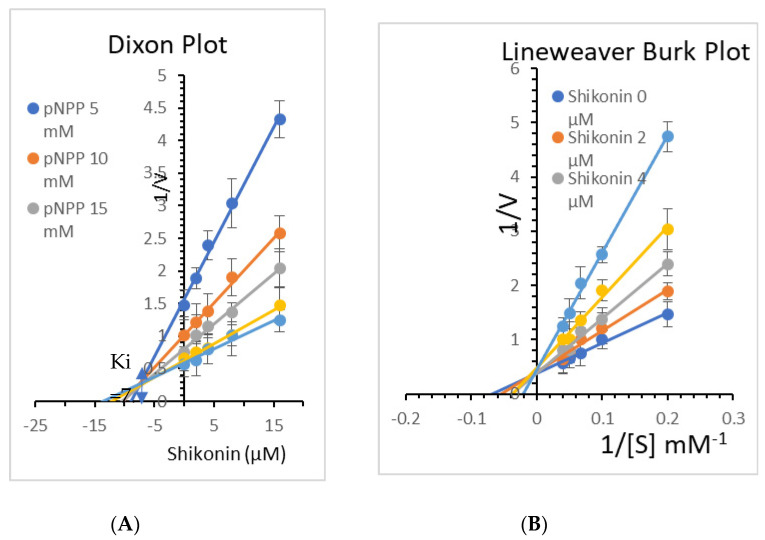
(**A**) Dixon plots of protein tyrosine phosphatases 1B (PTP1B) inhibition by Shikonin at various substrate (pNPP) concentrations (5, 10, 15, 20, and 25 mM). (**B**) Lineweaver–Burk plot for inhibition of PTP1B by Shikonin was analyzed in the presence of different concentrations of Shikonin (0, 2, 4, 8, and 16 µM).

Kinetic analysis was performed at different concentrations of the substrate (pNPP) and Shikonin to clarify the mode of enzyme PTP1B inhibition. Shikonin exhibited competitive inhibition against both PTPs (Ki = 7.5 M) (Figure 13). A lower Ki value can mean more effective inhibition against the enzyme system in the production of preventive and therapeutic agents because the Ki value represents the concentration required to form an enzyme-inhibitor complex.

## 4. Conclusions

The findings of our research conclude that the Shikonin (dye pigment and phytoconstituent) couldbe used as a potent hypoglycemic agent, as it inhibits PTP1B possibly at the catalytic domain. The ligands’ binding affinities to the target PTP1B active site were quantified using docking results and molecular dynamics simulations. The combined in silico studies identified structural modifications that could aid in the development of more successful PTP1B inhibitors through strategic molecular architecture design and optimization. The Shikonin scaffold and their antidiabetic effects as PTP1B inhibitors are discussed in depth in this article, providing new perspectives for the development of new drugs for the treatment of type 2 diabetes.

## Figures and Tables

**Table 1:** Computational parameters of pharmacokinetics (ADME) of Shikonin pharmacophores as calculated by the ADMET Descriptor.

Pharmacophore	AlogP	PSA_2D	Plasma Protein Binding	Hepatotoxicity	CYP2D6 Binding	Aqueous Solubility	BBB Penetration	Intestinal Absorption
Shikonin	2.444	97.048	True (highly bound)	True (toxic)	False (non-inhibitor)	3 (good)	3 (low)	0 (good)
ZINC31168041	3.725	79.747	False (poorly bounded)	False (non-toxic)	False (non-inhibitor)	3 (good)	4 (undefined)	1 (moderate)
ZINC31168045	3.725	118.422	False (poorly bounded)	False (non-toxic)	False (non-inhibitor)	3 (good)	4 (undefined)	1 (moderate)
ZINC31168048	3.725	118.422	False (poorly bounded)	False (non-toxic)	False (non-inhibitor)	3 (good)	4 (undefined)	1 (moderate)
ZINC31168554	1.039	118.422	False (poorly bounded)	True (toxic)	False (non-inhibitor)	4 (optimal)	3 (low)	0 (good)

Abbreviations: AlogP, the logarithm of the partition coefficient between n-octanol and water; PSA, polar surface area; BBB, blood-brain barrier.

**Table 2:** Computed drug-likeness for Shikonin and filtered compounds by the Lipinski and Veberrule.

Compounds	Jurs TPSA (<140 Å^2^)	MW (<500)	ALog P (≤5)	H-Bond Donor (≤5)	H-Bond Acceptor (≤10)	Rule of 5 Violations
Shikonin	164.344	288.295	2.444	3	5	1
ZINC31168041	192.687	432.507	3.725	4	7	1
ZINC31168045	218.542	432.507	3.725	4	7	1
ZINC31168048	186.02	432.507	3.725	4	7	1
ZINC31168554	162.648	196.2	1.039	3	4	1

Abbreviations: TPSA, topological polar surface area; MW, molecular weight; LogP = octanol/water partition coefficient.

**Table 3:** Computational values of toxicity and carcinogenicity predicted for Shikonin and top four filtered molecules by Biovia Discovery Studio.

Parameters	Shikonin	ZINC31168041	ZINC31168045	ZINC31168048	ZINC31168554
Rate of Oral LD_50_ (g/kg body weight)	1.33207	4.97354	4.97354	4.97354	1.84165
Rat inhalation LC_50_ (mg/m^3^/h)	2359.66	1288.65	1288.65	1288.65	7376.76
Daphnia EC_50_ (mg/mL)	25.4641	1.89946	1.89946	1.89946	506.316
Rat chronic LOEAL (g/kg body weight)	0.0825521	0.0370396	0.0370396	0.0370396	0.220851
Fathead minnow LC_50_ (g/L)	0.0185828	0.000967424	0.000967424	0.000967424	0.802847
**Carcinogenic potency TD_50_ (mg/kg body weight/day)**					
Mouse	132.774	176.288	176.288	176.288	339.579
Rat	25.1746	9.39947	9.39947	9.39947	7.86891
Rat maximum tolerated dose (g/kg body weight)	0.697318	0.938401	0.938401	0.938401	1.03762
Ames mutagenicity	Non-Mutagen	Non-Mutagen	Non-Mutagen	Non-Mutagen	Non-Mutagen
Developmental toxicity potential	Toxic	Toxic	Toxic	Toxic	Toxic
Aerobic biodegradability	Degradable	Degradable	Degradable	Degradable	Degradable
Ocular irritancy	None	None	None	None	Moderate
Skin irritancy	None	Mild	Mild	Mild	None

Abbreviations: LD_50_, lethal dose 50%; LC_50_, lethal concentration 50%; EC_50_, effective concentration 50%; LOAEL, lowest observed adverse effect level; TD_50_, tumorigenic dose 50%.

**Table 4:** Hydrogen bond interactions formed between the complexes of PTP1B-Shikonin, and PTP1B- top 4 filtered molecules within the active site.

S. No.	Compound	Hydrogen Bonds	Gold Fitness Score
1.	Shikonin	ARG24, GLN262, ASP48, MET258, AND SER28	46.3057
2.	ZINC31168041	SER215, SER216, ALA217, GLY218, ILE219, GLY220, ARG221, ARG254, ARG24, SER215, SER216, GLN262.	116.001
3.	ZINC31168045	ARG24, PHE182, SER215, SER216, ALA217, GLY220, ARG221, ARG254, GLN266.	118.114
4.	ZINC31168048	ARG24, PHE182, SER215, ALA217, GLY218, ILE219, ARG221, ARG254, GLN266, ASP181.	115.39
5.	ZINC31168554	LYS120, SER216, ARG221, GLN266, SER215	59.7591

## Data Availability

Not applicable.

## Supplementary Materials

The following are available online. Table S1: Computational parameters of pharmacokinetics (ADME) of Shikonin pharmacophores as calculated by ADMET Descriptor; Table S2: Computed computational values for Shikonin and its pharamacophores using Biovia Discovery Studio version 4.5.

## Sample Availability

Samples of the compounds are available from the authors.
