# Supplementary material for: Assessment of Antidiabetic Activity of the Shikonin by Allosteric Inhibition of Protein-Tyrosine Phosphatase 1B (PTP1B) Using State of Art: An In Silico and In Vitro Tactics
Source: Molecules. 2021 Jun 30;26(13):3996. doi: 10.3390/molecules26133996 (PMC8271486; doi:10.3390/molecules26133996)
Supplement: Supplementary file 1 [file molecules-26-03996-s001.zip › Table S1.pdf]

Table S1: Computational parameters of pharmacokinetics (ADME) of Shikonin pharmacophores as calculated by ADMET Descriptor.

Compounds	Aquous Solubility	BBB penetration	CYP2D6 Binding	Hepatotoxicity	Intestinal absorption	AlogP	PSA_2D
ZINC04252531	3	4	FALSE	FALSE	3	1.069	160.054
ZINC31154941	4	4	FALSE	FALSE	3	-1.851	181.428
ZINC35456730	3	4	FALSE	FALSE	3	0.444	168.984
ZINC03874669	3	4	FALSE	FALSE	3	0.827	180.869
ZINC31157223	3	4	FALSE	FALSE	3	1.08	168.984
ZINC13404388	3	4	FALSE	FALSE	3	0.04	201.684
ZINC31168395	3	4	FALSE	TRUE	3	-0.422	213.57
ZINC13404384	3	4	FALSE	FALSE	3	0.04	201.684
ZINC37538744	3	4	FALSE	FALSE	3	1.144	168.984
ZINC31155964	3	4	FALSE	FALSE	2	3.074	142.753
ZINC31155960	3	4	FALSE	FALSE	2	3.074	142.753
ZINC31155572	3	4	FALSE	TRUE	3	3.1	163.568
ZINC13424696	2	4	FALSE	FALSE	3	0.32	222.5
ZINC13404408	3	4	FALSE	FALSE	3	-0.202	222.5
ZINC35454596	4	4	FALSE	FALSE	3	-2.15	231.43
ZINC35454599	4	4	FALSE	FALSE	3	-2.15	231.43
ZINC35454588	4	4	FALSE	FALSE	3	-2.15	231.43
ZINC35454592	4	4	FALSE	FALSE	3	-2.15	231.43
ZINC04349223	2	4	FALSE	TRUE	3	-0.541	231.43
ZINC35455254	4	4	FALSE	FALSE	3	-1.762	198.729
ZINC35271371	3	4	FALSE	FALSE	3	-0.006	181.428
ZINC04887566	2	4	FALSE	TRUE	3	-0.052	248.731
ZINC04887568	2	4	FALSE	TRUE	3	-0.052	248.731
ZINC35455036	3	4	FALSE	TRUE	3	-1.923	216.03
ZINC31167460	3	4	FALSE	TRUE	3	-0.367	185.725
ZINC35464471	3	4	FALSE	TRUE	3	-0.367	185.725
ZINC31167451	3	4	FALSE	TRUE	3	-0.367	185.725
ZINC31167448	3	4	FALSE	TRUE	3	-0.367	185.725
ZINC31167456	3	4	FALSE	TRUE	3	-0.367	185.725
ZINC67912024	3	4	FALSE	FALSE	3	-1.131	198.729
ZINC13451185	3	4	FALSE	FALSE	3	0.213	211.956
ZINC72320586	3	4	FALSE	FALSE	3	-0.504	211.956
ZINC72320588	3	4	FALSE	FALSE	3	-0.504	211.956
ZINC67902596	2	4	FALSE	FALSE	3	-3.148	261.176
ZINC72320007	3	4	FALSE	FALSE	3	-2.058	228.475
ZINC35455227	3	4	FALSE	FALSE	3	1.096	181.428
ZINC35455235	3	4	FALSE	FALSE	3	1.096	181.428
ZINC67912601	3	4	FALSE	FALSE	3	-2.148	233.331

ZINC67912606	3	4	FALSE	FALSE	3	-2.148	233.331
ZINC67913702	4	4	FALSE	FALSE	3	-2.405	212.515
ZINC35465647	3	4	FALSE	FALSE	3	4.253	160.054
ZINC72320594	3	4	FALSE	FALSE	3	-0.513	190.358
ZINC72320595	3	4	FALSE	FALSE	3	-0.513	190.358
ZINC72320597	3	4	FALSE	FALSE	3	-0.513	190.358
ZINC72320596	3	4	FALSE	FALSE	3	-0.513	190.358
ZINC67910363	3	4	FALSE	FALSE	3	-0.199	190.358
ZINC38143583	3	4	FALSE	FALSE	3	-0.448	224.96
ZINC38143581	3	4	FALSE	FALSE	3	-0.448	224.96
ZINC85341116	3	4	FALSE	FALSE	3	-0.448	224.96
ZINC38143580	3	4	FALSE	FALSE	3	-0.448	224.96
ZINC67912507	3	4	FALSE	FALSE	3	-0.525	204.145
ZINC67910645	4	4	FALSE	FALSE	3	-2.532	238.746
ZINC85340579	2	4	FALSE	TRUE	3	-0.211	228.475
ZINC77257082	2	4	FALSE	TRUE	3	-1.689	257.661
ZINC38139587	3	4	FALSE	TRUE	3	0.517	199.289
ZINC67902744	2	4	FALSE	FALSE	3	-1.317	249.29
ZINC67912012	3	4	FALSE	FALSE	3	-1.987	245.776
ZINC33861425	2	4	FALSE	FALSE	3	2.144	248.731
ZINC67902892	2	4	FALSE	TRUE	3	-0.62	249.29
ZINC67913374	3	4	FALSE	FALSE	3	1.665	202.244
ZINC70672772	3	4	FALSE	TRUE	3	-0.303	224.35
ZINC72320143	2	4	FALSE	TRUE	3	6.988	159.494
ZINC72320145	2	4	FALSE	TRUE	3	6.988	159.494
ZINC72320142	2	4	FALSE	TRUE	3	6.988	159.494
ZINC67903086	2	4	FALSE	TRUE	3	-0.657	249.29
ZINC59816865	2	4	FALSE	TRUE	3	-1.536	270.106
ZINC70691533	2	4	FALSE	TRUE	3	-1.536	270.106
ZINC08234294	2	4	FALSE	TRUE	3	-0.657	249.29
ZINC67910222	3	4	FALSE	FALSE	3	2.215	202.244
ZINC08143568	2	4	FALSE	TRUE	3	-0.431	237.405
ZINC67912005	2	4	FALSE	FALSE	3	0.106	249.29
ZINC67902708	2	4	FALSE	TRUE	3	5.898	180.31
ZINC67902702	2	4	FALSE	TRUE	3	5.898	180.31
ZINC38143675	2	4	FALSE	FALSE	3	-1.334	258.22
ZINC38143673	2	4	FALSE	FALSE	3	-1.334	258.22
ZINC38143676	2	4	FALSE	FALSE	3	-1.334	258.22
ZINC38143674	2	4	FALSE	FALSE	3	-1.334	258.22
ZINC67902872	2	4	FALSE	FALSE	3	0.484	249.29
ZINC49898792	2	4	FALSE	FALSE	3	0.484	249.29

ZINC08234345	2	4	FALSE	FALSE	3	0.484	249.29
ZINC67902876	1	4	FALSE	FALSE	3	-2.046	290.921
ZINC67912153	2	4	FALSE	FALSE	3	-0.726	270.106
ZINC49781425	1	4	FALSE	TRUE	3	-2.229	290.921
ZINC33861449	0	4	FALSE	TRUE	3	0.776	299.292
ZINC67910683	2	4	FALSE	FALSE	3	-1.569	267.151
ZINC67910687	2	4	FALSE	FALSE	3	-1.569	267.151
ZINC67910690	2	4	FALSE	FALSE	3	-1.569	267.151
ZINC67902500	3	4	FALSE	FALSE	3	-0.62	243.38
ZINC67911949	3	4	FALSE	FALSE	3	1.103	239.306
ZINC77269667	2	4	FALSE	FALSE	3	1.666	257.661
ZINC67910405	1	4	FALSE	TRUE	3	1.567	288.188
ZINC67903541	2	4	FALSE	TRUE	3	1.372	288.188
ZINC67903538	2	4	FALSE	TRUE	3	1.372	288.188
ZINC79210091	2	4	FALSE	TRUE	3	1.507	263.007
ZINC79210094	2	4	FALSE	TRUE	3	1.507	263.007
ZINC67902657	2	4	FALSE	FALSE	3	0.256	261.735
