# Supplementary material for: Assessment of Antidiabetic Activity of the Shikonin by Allosteric Inhibition of Protein-Tyrosine Phosphatase 1B (PTP1B) Using State of Art: An In Silico and In Vitro Tactics
Source: Molecules. 2021 Jun 30;26(13):3996. doi: 10.3390/molecules26133996 (PMC8271486; doi:10.3390/molecules26133996)
Supplement: Supplementary file 1 [file molecules-26-03996-s001.zip › Table S2.pdf]

Table S2: Computed computational values for Shikonin and its pharamacophores using Biovia Discovery Studio version 4.5

Compounds	Carcinogenic potency TD50_Mouse (mg/kg body weight/day)	Carcinogenic potency TD50_Rat (mg/kg body weight/day)	Ames mutagenicity	Develop mental toxicity potential	Rate of Oral LD50 (g/kg body weight)	Rat maximum tolerated dose (g/kg body weight)	Rat inhalation LC50 (mg/m3/h)	Rat chronic LOEAL (g/kg body weight)	Skin irritancy	Ocular irritancy	Aerobic biodegrad ability	Fathead minnow LC50 (g/L)	Daphnia _EC50
ZINC04252531	69.6814	58.8549	Non-Mutagen	Toxic	1.34461	1.60205	128.234	0.119915	None	Mild	Degradable	0.083727	9.15815
ZINC31154941	17.6526	8.70416	Non-Mutagen	Toxic	2.99297	1.54515	40.8136	0.047911	Mild	Severe	Non-Degradable	10.037	5.54091
ZINC35456730	26.9648	4.35973	Non-Mutagen	Toxic	1.43456	1.02705	246.749	0.058777	None	Severe	Degradable	0.274372	12.58
ZINC03874669	22.49	10.8606	Non-Mutagen	Toxic	5.90786	2.41508	79.396	0.091103	None	Severe	Degradable	0.150494	7.57431
ZINC31157223	79.0705	48.1081	Non-Mutagen	Toxic	4.94696	1.20099	99.0829	0.05374	None	Mild	Degradable	0.008355	1.50118
ZINC13404388	10.0204	13.6469	Non-Mutagen	Toxic	1.90891	2.55866	59.2914	0.103979	None	Severe	Degradable	0.315966	17.2369
ZINC31168395	7.81565	8.08092	Non-Mutagen	Toxic	2.27186	4.5905	73.161	0.170122	None	Severe	Degradable	1.17299	33.0082
ZINC13404384	10.0204	13.6469	Non-Mutagen	Toxic	1.90891	2.55866	59.2914	0.103979	None	Severe	Degradable	0.315966	17.2369
ZINC37538744	20.3629	7.15743	Non-Mutagen	Toxic	4.37715	0.861081	134.88	0.030308	None	Severe	Degradable	0.008699	2.19146

ZINC31155964	12.5847	20.6939	Non-Mutagen	Toxic	3.77256	2.39203	147.423	0.175244	None	Severe	Non-Degradable	0.007458	0.32656
ZINC31155960	12.5847	20.6939	Non-Mutagen	Toxic	3.77256	2.39203	147.423	0.175244	None	Severe	Non-Degradable	0.007458	0.32656
ZINC31155572	17.0777	3.50331	Non-Mutagen	Toxic	8.02132	3.84466	226.858	0.228746	None	Severe	Non-Degradable	0.040068	0.393623
ZINC13424696	11.0715	26.8242	Non-Mutagen	Toxic	2.50058	3.8423	26.3121	0.043173	None	Mild	Degradable	0.116181	4.93771
ZINC13404408	12.0324	11.972	Non-Mutagen	Toxic	2.8932	3.84715	55.8623	0.149216	None	Severe	Degradable	0.566452	12.5948
ZINC35454596	69.3455	27.1667	Non-Mutagen	Toxic	10.7402	5.26155	21.8685	0.108787	Mild	Moderate	Degradable	1.73957	4.62572
ZINC35454599	69.3455	27.1667	Non-Mutagen	Toxic	10.7402	5.26155	21.8685	0.108787	Mild	Moderate	Degradable	1.73957	4.62572
ZINC35454588	69.3455	27.1667	Non-Mutagen	Toxic	10.7402	5.26155	21.8685	0.108787	Mild	Moderate	Degradable	1.73957	4.62572
ZINC35454592	69.3455	27.1667	Non-Mutagen	Toxic	10.7402	5.26155	21.8685	0.108787	Mild	Moderate	Degradable	1.73957	4.62572
ZINC04349223	7.34201	3.97989	Mutagen	Toxic	1.62863	3.47307	37.364	0.0999	None	Moderate	Degradable	1.38391	4.15102
ZINC35455254	9.65831	42.967	Non-Mutagen	Toxic	0.694201	1.07197	17.7249	0.010674	Mild	None	Degradable	0.305931	5.67392
ZINC35271371	25.0999	2.76499	Non-Mutagen	Toxic	3.81898	0.840288	246.447	0.073495	Mild	Severe	Degradable	0.210485	2.52601
ZINC04887566	8.13962	0.941567	Non-Mutagen	Toxic	3.17515	6.35874	26.4469	0.070434	None	None	Degradable	1.55141	1.94

ZINC04887568	8.13962	0.941567	Non-Mutagen	Toxic	3.17515	6.35874	26.4469	0.070434	None	None	Degradable	1.55141	1.94
ZINC35455036	75.9842	1.11727	Non-Mutagen	Toxic	3.29896	0.774151	253.108	0.055891	Mild	None	Degradable	1.43767	13.9051
ZINC31167460	65.8207	9.01344	Non-Mutagen	Toxic	1.38715	1.36179	68.5541	0.053914	None	Mild	Non-Degradable	0.263736	17.2781
ZINC35464471	65.8207	9.01344	Non-Mutagen	Toxic	1.38715	1.36179	68.5541	0.053914	None	Mild	Non-Degradable	0.263736	17.2781
ZINC31167451	65.8207	9.01344	Non-Mutagen	Toxic	1.38715	1.36179	68.5541	0.053914	None	Mild	Non-Degradable	0.263736	17.2781
ZINC31167448	65.8207	9.01344	Non-Mutagen	Toxic	1.38715	1.36179	68.5541	0.053914	None	Mild	Non-Degradable	0.263736	17.2781
ZINC31167456	65.8207	9.01344	Non-Mutagen	Toxic	1.38715	1.36179	68.5541	0.053914	None	Mild	Non-Degradable	0.263736	17.2781
ZINC67912024	8.40892	4.21256	Non-Mutagen	Toxic	4.24621	1.14774	41.7276	0.058482	Mild	Severe	Degradable	0.608756	3.9021
ZINC13451185	92.4644	4.39451	Non-Mutagen	Toxic	1.40307	0.9538	19.035	0.021904	None	Mild	Degradable	0.034027	0.650872
ZINC72320586	575.771	14.0133	Non-Mutagen	Toxic	15.4018	0.385415	140.479	0.308132	None	None	Degradable	0.170306	6.53117
ZINC72320588	575.771	14.0133	Non-Mutagen	Toxic	15.4018	0.385415	140.479	0.308132	None	None	Degradable	0.170306	6.53117
ZINC67902596	3.98104	3.98096	Mutagen	Toxic	2.2648	1.96041	7.70972	0.096909	Mild	Severe	Degradable	5.42036	7.09464

ZINC72320007	28.7861	3.70919	Non-Mutagen	Toxic	10.8624	1.11336	24.1738	0.030159	Mild	Moderate	Degradable	0.612987	2.71009
ZINC35455227	18.5022	2.51041	Non-Mutagen	Toxic	15.6244	1.07101	148.967	0.118608	Mild	Severe	Degradable	0.023542	0.508021
ZINC35455235	18.5022	2.51041	Non-Mutagen	Toxic	15.6244	1.07101	148.967	0.118608	Mild	Severe	Degradable	0.023542	0.508021
ZINC67912601	20.9066	32.2917	Non-Mutagen	Toxic	2.41083	1.41083	22.4275	0.059894	Mild	None	Degradable	0.179717	4.63261
ZINC67912606	20.9066	32.2917	Non-Mutagen	Toxic	2.41083	1.41083	22.4275	0.059894	Mild	None	Degradable	0.179717	4.63261
ZINC67913702	15.2265	23.2325	Non-Mutagen	Toxic	0.434574	0.459913	8.82423	0.007355	Mild	Moderate	Degradable	0.280791	2.74662
ZINC35465647	48.183	161.257	Non-Mutagen	Toxic	3.20907	1.69036	111.934	0.045535	Moderate	None	Degradable	0.000121	0.419765
ZINC72320594	14.2565	1.82097	Non-Mutagen	Toxic	8.01423	0.573214	199.942	0.082339	Mild	Severe	Degradable	0.224036	1.56435
ZINC72320595	14.2565	1.82097	Non-Mutagen	Toxic	8.01423	0.573214	199.942	0.082339	Mild	Severe	Degradable	0.224036	1.56435
ZINC72320597	14.2565	1.82097	Non-Mutagen	Toxic	8.01423	0.573214	199.942	0.082339	Mild	Severe	Degradable	0.224036	1.56435
ZINC72320596	14.2565	1.82097	Non-Mutagen	Toxic	8.01423	0.573214	199.942	0.082339	Mild	Severe	Degradable	0.224036	1.56435
ZINC67910363	7.93947	1.81409	Non-Mutagen	Toxic	5.1196	0.605959	86.0014	0.038287	Mild	Severe	Degradable	0.155868	1.23559
ZINC38143583	16.2005	2.12237	Non-Mutagen	Toxic	5.2689	1.36735	62.1922	0.037211	Mild	None	Degradable	0.008392	2.26367
ZINC38143581	16.2005	2.12237	Non-Mutagen	Toxic	5.2689	1.36735	62.1922	0.037211	Mild	None	Degradable	0.008392	2.26367
ZINC85341116	16.2005	2.12237	Non-Mutagen	Toxic	5.2689	1.36735	62.1922	0.037211	Mild	None	Degradable	0.008392	2.26367

ZINC38143580	16.2005	2.12237	Non-Mutagen	Toxic	5.2689	1.36735	62.1922	0.037211	Mild	None	Degradable	0.008392	2.26367
ZINC67912507	11.6043	3.41078	Non-Mutagen	Toxic	1.10185	0.387106	23.7301	0.012693	Mild	None	Degradable	0.01741	2.575
ZINC67910645	87.2611	3.80584	Non-Mutagen	Toxic	11.4092	0.525397	19.1937	0.161848	Mild	Mild	Degradable	0.578979	1.57282
ZINC85340579	5.13669	0.429544	Non-Mutagen	Toxic	2.35208	0.791358	36.9411	0.084906	None	Moderate	Degradable	0.78033	0.495827
ZINC77257082	5.3455	0.578995	Non-Mutagen	Toxic	7.52439	1.21209	8.65411	0.11207	None	Mild	Non-Degradable	4.1767	1.80417
ZINC38139587	6.55699	0.487204	Non-Mutagen	Toxic	1.70472	0.566447	87.7859	0.044118	Mild	Mild	Degradable	0.049654	0.44407
ZINC67902744	18.294	15.6934	Non-Mutagen	Toxic	8.33225	2.10688	5.14746	0.222251	None	None	Degradable	0.661296	1.81436
ZINC67912012	14.5437	2.958	Non-Mutagen	Toxic	4.33803	0.861826	13.0763	0.030017	Mild	None	Degradable	0.162476	1.89258
ZINC33861425	2.91834	0.646121	Non-Mutagen	Toxic	27.1477	5.04098	12.598	0.145151	None	None	Degradable	0.033904	0.557349
ZINC67902892	2.81057	0.848466	Non-Mutagen	Toxic	3.24553	1.43267	22.6831	0.039401	None	Moderate	Degradable	0.79128	1.09705
ZINC67913374	4.54224	6.73813	Non-Mutagen	Toxic	4.06941	3.45435	19.3757	0.089729	Mild	Severe	Non-Degradable	0.04343	0.103406
ZINC70672772	68.8985	21.3189	Non-Mutagen	Toxic	4.60177	3.69439	9.0042	0.219997	None	Moderate	Degradable	0.029333	0.304376
ZINC72320143	24.8795	3.44004	Non-Mutagen	Toxic	1.72121	1.58236	246.937	0.076542	Moderate	None	Degradable	1.21E-05	0.104321
ZINC72320145	24.8795	3.44004	Non-Mutagen	Toxic	1.72121	1.58236	246.937	0.076542	Moderate	None	Degradable	1.21E-05	0.104321

ZINC72320142	24.8795	3.44004	Non-Mutagen	Toxic	1.72121	1.58236	246.937	0.076542	Moderate	None	Degradable	1.21E-05	0.104321
ZINC67903086	4.05087	0.243604	Non-Mutagen	Toxic	5.91796	1.7991	29.3084	0.081602	None	Mild	Degradable	4.08273	2.58184
ZINC59816865	3.31067	0.359775	Non-Mutagen	Toxic	5.0698	2.73912	9.01174	0.054298	None	Moderate	Degradable	7.30341	0.513099
ZINC70691533	3.31067	0.359775	Non-Mutagen	Toxic	5.0698	2.73912	9.01174	0.054298	None	Moderate	Degradable	7.30341	0.513099
ZINC08234294	4.05087	0.243604	Non-Mutagen	Toxic	5.91796	1.7991	29.3084	0.081602	None	Mild	Degradable	4.08273	2.58184
ZINC67910222	3.27614	1.37765	Non-Mutagen	Toxic	7.42822	2.76496	33.0286	0.265995	None	Severe	Non-Degradable	0.06142	0.139358
ZINC08143568	6.05684	0.0509088	Non-Mutagen	Toxic	2.89041	0.764033	39.6325	0.050189	None	Mild	Degradable	1.56319	1.71086
ZINC67912005	6.19456	0.812267	Non-Mutagen	Toxic	8.71191	3.10306	7.81091	0.046176	Mild	Mild	Degradable	0.093304	0.325256
ZINC67902708	22.8599	12.7737	Non-Mutagen	Toxic	1.78829	2.54931	120.637	0.11033	Moderate	None	Degradable	2.76E-05	0.214567
ZINC67902702	22.8599	12.7737	Non-Mutagen	Toxic	1.78829	2.54931	120.637	0.11033	Moderate	None	Degradable	2.76E-05	0.214567
ZINC38143675	17.0195	2.29434	Non-Mutagen	Toxic	20.9397	1.33449	8.11417	0.196993	None	None	Degradable	0.450839	1.95655
ZINC38143673	17.0195	2.29434	Non-Mutagen	Toxic	20.9397	1.33449	8.11417	0.196993	None	None	Degradable	0.450839	1.95655
ZINC38143676	17.0195	2.29434	Non-Mutagen	Toxic	20.9397	1.33449	8.11417	0.196993	None	None	Degradable	0.450839	1.95655
ZINC38143674	17.0195	2.29434	Non-Mutagen	Toxic	20.9397	1.33449	8.11417	0.196993	None	None	Degradable	0.450839	1.95655

ZINC67902872	4.06204	0.646873	Non-Mutagen	Toxic	7.55963	3.02237	13.5263	0.077166	Mild	Severe	Degradable	0.081162	0.556794
ZINC49898792	4.06204	0.646873	Non-Mutagen	Toxic	7.55963	3.02237	13.5263	0.077166	Mild	Severe	Degradable	0.081162	0.556794
ZINC08234345	4.06204	0.646873	Non-Mutagen	Toxic	12.9722	3.02237	11.1012	0.08879	Mild	Mild	Degradable	0.081162	0.526095
ZINC67902876	2.53636	1.6954	Non-Mutagen	Toxic	2.40562	2.87937	3.5463	0.120496	None	Moderat e	Degradable	4.29934	0.635534
ZINC67912153	7.13047	0.778172	Non-Mutagen	Toxic	9.45009	4.16567	8.06655	0.042773	Mild	Severe	Degradable	0.321934	0.519982
ZINC49781425	2.78678	2.18562	Non-Mutagen	Toxic	2.74898	2.78764	3.484	0.110585	None	Moderat e	Degradable	5.2484	0.727117
ZINC33861449	0.884806	0.139494	Non-Mutagen	Toxic	1.88032	5.3752	9.96899	0.107901	None	None	Degradable	0.304395	0.600581
ZINC67910683	4.77223	0.286416	Non-Mutagen	Toxic	4.65276	0.864701	11.648	0.06716	None	Mild	Degradable	1.30378	0.513892
ZINC67910687	4.77223	0.286416	Non-Mutagen	Toxic	4.65276	0.864701	11.648	0.06716	None	Mild	Degradable	1.30378	0.513892
ZINC67910690	4.77223	0.286416	Non-Mutagen	Toxic	4.65276	0.864701	11.648	0.06716	None	Mild	Degradable	1.30378	0.513892
ZINC67902500	6.9208	0.16944	Non-Mutagen	Toxic	4.17245	0.606871	25.6766	0.016362	Mild	None	Degradable	0.206598	0.35914
ZINC67911949	11.8383	3.85487	Non-Mutagen	Toxic	7.20504	0.505048	12.5004	0.047896	Mild	None	Degradable	0.00033	0.070361 4
ZINC77269667	4.38983	3.8934	Non-Mutagen	Toxic	7.2801	2.58198	13.4248	0.03111	Mild	Severe	Degradable	0.0022	0.19497
ZINC67910405	4.96145	15.7168	Non-Mutagen	Toxic	12.6361	1.31412	4.37969	0.184455	Mild	Mild	Non-Degradable	0.000267	0.011086

ZINC67903541	5.9924	7.3021	Non-Mutagen	Toxic	4.78204	1.39205	3.85974	0.301067	None	Moderate	Non-Degradable	0.000313	0.0200298
ZINC67903538	5.9924	7.3021	Non-Mutagen	Toxic	4.78204	1.39205	3.85974	0.301067	None	Moderate	Non-Degradable	0.000313	0.0200298
ZINC79210091	5.99396	0.382049	Non-Mutagen	Toxic	2.39611	4.10972	6.881	0.129576	None	Mild	Degradable	0.003293	0.0164177
ZINC79210094	5.99396	0.382049	Non-Mutagen	Toxic	2.39611	4.10972	6.881	0.129576	None	Mild	Degradable	0.003293	0.0164177
ZINC67902657	1.53623	2.6866	Non-Mutagen	Toxic	7.50652	4.2508	1.97767	0.120862	Mild	Severe	Non-Degradable	0.136052	0.0278864
